# Supplementary material for: Evaluation of Pre-Applied Conductive Materials in Electrode Grids for Longterm EEG Recording
Source: Sensors (Basel). 2025 Nov 7;25(22):6810. doi: 10.3390/s25226810 (PMC12656683; doi:10.3390/s25226810)
Supplement: Supplementary file 1 [file sensors-25-06810-s001.zip › sensors-3925627-supplementary.pdf]

Supplementary Materials

Evaluation of Pre-Applied Conductive Materials in Electrode Grids for long-term EEG recording

Comparison of new materials to standard ring electrodes

To compare the two materials used in this study (hydrogel and silicone) to standard Ag/AgCl ring electrodes we collected an additional, small EEG dataset (N=2) test to record continuous EEG data. We conducted two separate measurements each with one of the two participants: 1. Standard ring electrodes vs. silicone and 2. standard ring electrode vs. hydrogel.

Materials and Methods

The test procedures were identical each time. Three ring electrodes were used. Two were placed behind one ear to serve as the reference and ground electrodes, and one was placed on the on the forehead (Ring – R1). The skin was prepared with alcohol, and the ring electrodes were filled with standard EEG electrolyte gel.
For the silicone comparison, two flex-printed electrode stripes prepared with silicone were used. Two electrodes were placed behind one ear as the reference and ground, and one was placed on the forehead (Silicone—Sil1) next to the ring electrode. The skin sites were prepared with alcohol as described in the paper (see Fig S1. for electrode positions).
For the hydrogel comparison, two flex-printed electrode stripes prepared with hydrogel were used. Two electrodes were placed behind one ear as the reference and ground, and one was placed on the forehead (Hydrogel—Hyd1) next to the ring electrode. The skin sites were prepared with alcohol as described in the paper (see Fig S2. for electrode positions).
Each of the electrode systems was connected to a separate amplifier and recorded synchronously using LabStreamerProtocol, as described in the paper. The same auditory test as described in the Materials and Methods section was conducted.

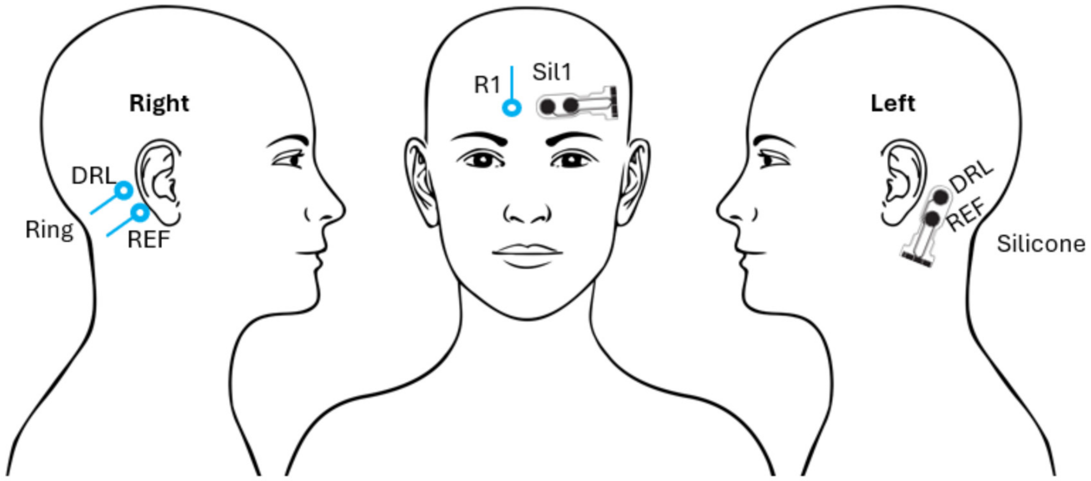

Figure **S1.** Electrode positions for the silicone–standard electrode comparison.

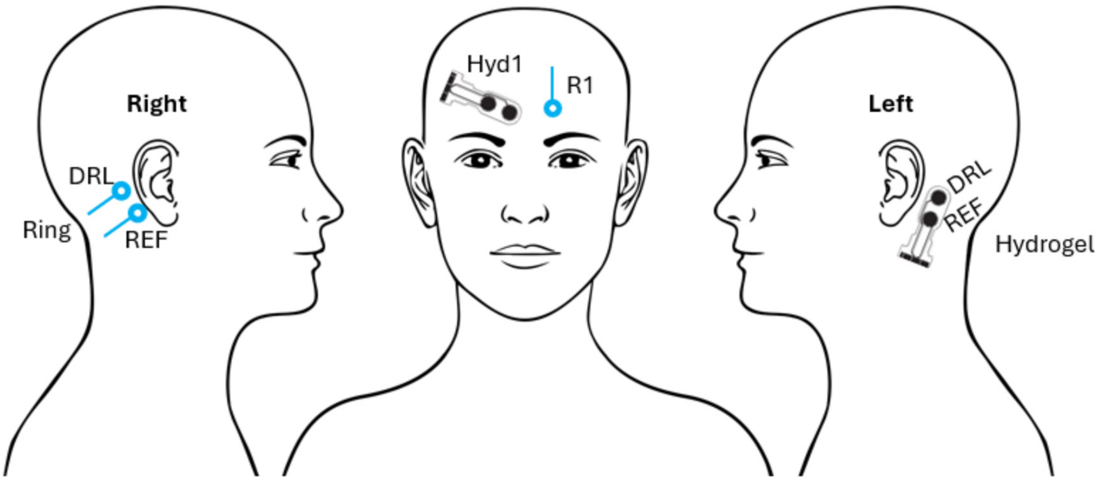

Figure S2. Electrode positions for the hydrogel–standard electrode comparison.

The EEG data was preprocessed as described in Section 2.3.4 of the main paper. EEG data were first filtered using a zero-phase, fourth-order Butterworth filter with a stop band between 45 and 55 Hz to remove line noise, and a second stop band between 60 and 65 Hz to remove noise caused by the impedance current. The data were then bandpass filtered (zero-phase, fourth-order Butterworth) between 0.5 and 20 Hz to remove drift and high-frequency noise.
To understand the relationship between state-of-the-art Ag/AgCl ring electrodes and the new materials correlational analyses were conducted on EEG voltage time series data. Separate Pearson r correlations were computed for Ring vs. Hydrogel and Ring vs. Silicone. Analyses were conducted on the entire time window of 12 minutes, as well as on a shorter time window of 30 and 6 seconds selected randomly from the entire dataset.

Results

Table S1 provides an overview of the Pearson r correlational values as well as the corresponding level of significance.
Table S1. Pearson r correlation values related to the comparisons between standard Ag/AgCl ring electrodes and each of the new materials (Hydrogel, Silicone) for three different lengths of EEG time series data sections.

	**Ring and Hydrogel**	**Ring and Silicone**
**12 minutes**	$r = 0.88$, $p < .001$	$r = 0.83$, $p < .001$
**30 seconds**	$r = 0.96$, $p < .001$	$r = 0.88$, $p < .001$
**6 seconds**	$r = 0.70$, $p < .001$	$r = 0.59$, $p < .001$

The figures below show the continuous EEG time series data for hydrogel for 30 seconds (Fig S3) and 6 seconds (Fig. S4), as well as for silicone for 30 seconds (Fig. S5) and 6 seconds (Fig. S6), always in comparison to a ring electrode.

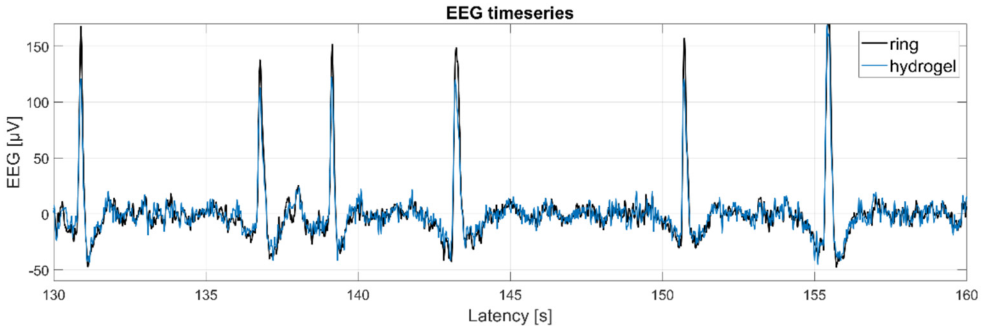

Figure S3. A 30-second window of EEG recorded from two channels on the forehead, referencing the mastoids. One channel was recorded with a standard Ag/AgCl ring electrode filled with electrolyte gel; the other channel was recorded with a flat flexprinted Ag/AgCl electrode covered with hydrogel. Eye blinks are clearly visible in the signal.

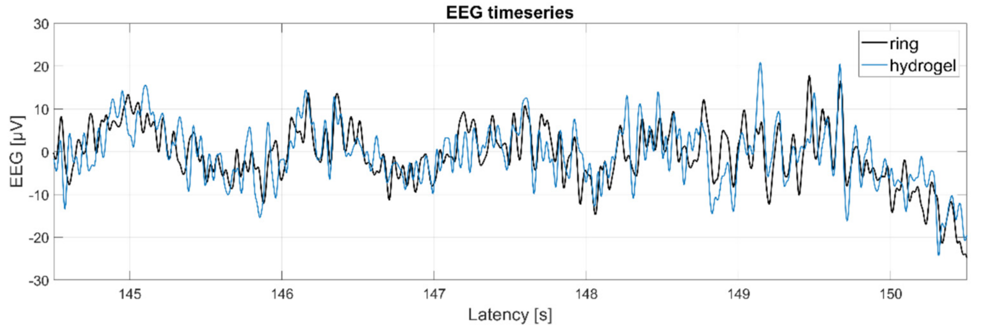

Figure S4. A 6-second window of EEG recorded from two channels on the forehead, referencing the mastoids. One channel was recorded with a standard Ag/AgCl ring electrode filled with electrolyte gel; the other channel was recorded with a flat flexprinted Ag/AgCl electrode covered with hydrogel.

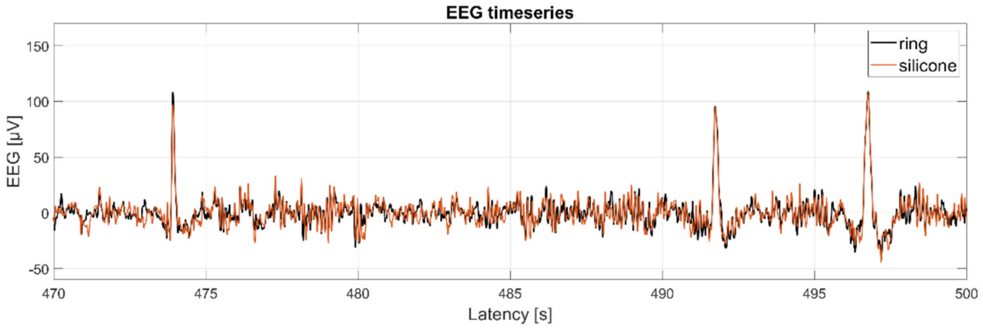

Figure S5. A 30-second window of EEG recorded from two channels on the forehead, referencinf to the mastoids. One channel was recorded with a standard Ag/AgCl ring electrode filled with electrolyte gel; the other channel was recorded with a flat flexprinted Ag/AgCl electrode covered with silicone. Eye blinks are clearly visible in the signal.

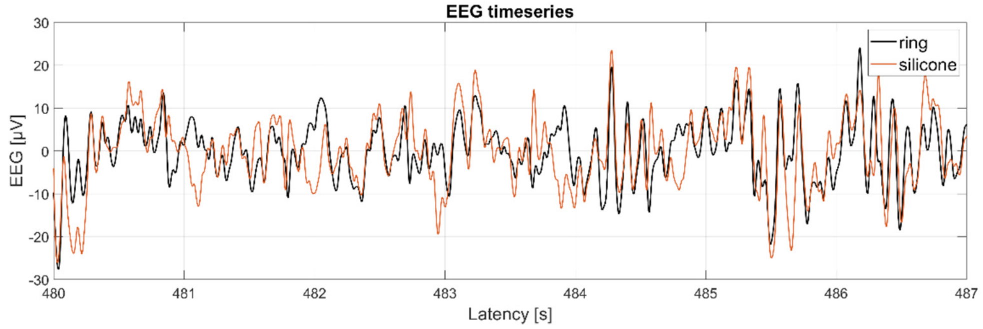

Figure S6. A 6-second window of EEG recorded from two channels on the forehead, referencing to the mastoids. One channel was recorded with a standard Ag/AgCl ring electrode filled with electrolyte gel; the other channel was recorded with a flat flexprinted Ag/AgCl electrode covered with silicone material.

Discussion

Time series of both silicone and hydrogel electrodes correlate highly with those of ring electrodes recorded at the same time. Note that ring electrodes and the electrodes with the new materials were connected to separate amplifiers. Also, even though the electrodes were placed near to each other on the forehead, the positions were not identical. Also, the position of the reference and ground electrodes differed for the ring electrode setup (right mastoid) and the new materials setup (left mastoid). These factors could lead to correlation values lower than 1.
